# A case–control study on egg consumption and risk of stroke among Iranian population

**DOI:** 10.1186/s41043-017-0104-2

**Published:** 2017-06-05

**Authors:** Roohallah Fallah-Moshkani, Mohammad Saadatnia, Forough Shakeri, Ammar Hassanzadeh Keshteli, Parvane Saneei, Bagher Larijani, Ahmad Esmaillzadeh

**Affiliations:** 1 Students’ Research Committee, Food Security Research Center, Department of Community Nutrition, School of Nutrition and Food Science, Isfahan University of Medical Sciences, Isfahan, Iran; 2 Isfahan Neuroscience Research Center, Isfahan University of Medical Sciences, Isfahan, Iran; 3 Integrative Functional Gastroenterology Research Center, Isfahan University of Medical Sciences, Isfahan, Iran; 4 Department of Medicine, University of Alberta, Edmonton, Canada; 5 Endocrinology and Metabolism Research Center, Endocrinology and Metabolism Clinical Sciences Institute, Tehran University of Medical Sciences, Tehran, Iran; 6 Obesity and Eating Habits Research Center, Endocrinology and Metabolism Molecular Cellular Sciences Institute, Tehran University of Medical Sciences, Tehran, Iran; 7 Department of Community Nutrition, School of Nutritional Sciences and Dietetics, Tehran University of Medical Sciences, PO Box 14155-6117, Tehran, Iran

**Keywords:** Egg consumption, Stroke, Diet, Iran

## Abstract

**Background:**

Most available data that linked intake of egg to risk of stroke came from western countries, with conflicting findings. We aimed to examine the association between egg consumption and risk of stroke among Iranian adults.

**Methods:**

In a hospital-based case–control study, 195 stroke patients, hospitalized in Alzahra University Hospital, were selected as cases and 195 control subjects, from patients hospitalized in other wards with no history of cerebrovascular diseases or neurologic disorders, were recruited. A validated 168-item food frequency questionnaire (FFQ) was used to assess participants’ usual dietary intake, including egg consumption, over the previous year. Other required information was gathered by the use of questionnaires.

**Results:**

Consumption of eggs was associated with lower odds of stroke, such that after adjustment for potential confounders, those in the highest category of egg intake (>2 eggs/week) were 77% lower odds to have stroke, compared with those with the lowest category of egg intake (<1 egg/week) (OR 0.23; 95% CI 0.11–0.45). Further controlling for body mass index strengthened the association (OR 0.20; 95% CI 0.09–0.41).

**Conclusions:**

We found evidence indicating that high intake of eggs (>2 eggs/week) during the past 1 year was associated with a lower risk of stroke. Further prospective studies are required to confirm these findings.

## Background

Stroke is a leading cause of disability worldwide [1–3]. There is a large geographic variation in stroke incidence and mortality around the world. Furthermore, most stroke deaths occur in developing countries, and the proportion is likely to increase with epidemiologic transition and the aging population. In the USA, one in 19 deaths per year is due to stroke [4]. It imposes great burden to the healthcare system; for instance, 36.5 billion dollars annually are spent for this condition in the USA [4]. The rate of stroke in Iran is one in 969 deaths per year [1]; this rate is comparable to that in Arab countries, higher than in sub-Saharan Africa, but lower than in developed countries [1].

Several factors can contribute to the incidence of stroke, and among them, lifestyle-related factors, including dietary intakes, are the most studied [1, 4]. The association of stroke with dietary intakes, hypertension, glucose intolerance, obesity, high serum cholesterol, smoking, and physical inactivity has been reported [1, 4]. However, limited data are available linking egg consumption to the risk of stroke. Eggs are an inexpensive and low-calorie source of many nutrients, including animal protein; essential amino acids; unsaturated fatty acids; vitamins D, E, B_12_; lutein; and zeaxanthin [5–7]. Previous studies have examined egg consumption in relation to serum lipid levels [8], preventing age-related macular degeneration (AMD) development, cataract [6], macular pigment optical density (MPOD) [9], risk of coronary heart disease (CHD), diabetes [7, 10], cancer [11], neural tube defects (NTD), and sarcopenia [12, 13]. In addition, several studies have reported no significant association between egg consumption and risk of stroke [7, 14–16]. However, some other investigators have indicated that animal products like egg were inversely associated with risk of stroke mortality [17].

It must be kept in mind that most available data that linked egg consumption to the risk of stroke came from western countries and limited data exist in this regard from Middle Eastern nations. Given the significant differences in lifestyle, dietary intakes, socioeconomic status, social and cultural factors between developing nations and western countries, and examining the association between egg consumption and risk of stroke in Middle Eastern populations might provide additional information. Although eggs are cheap sources of animal protein, interestingly, consumption of eggs in Middle Eastern populations was lower than in the USA and Europe [7, 18]. On the other hand, the rate of stroke is also lower in these countries than those in the western nations. Therefore, along with other lifestyle factors, egg consumption might also contribute to this difference. Therefore, given the conflicting available data, this study was done to examine the association between egg consumption and risk of stroke among Iranian adults.

## Methods

### Participants

This hospital-based case–control study was done in Alzahra University Hospital in Isfahan, Iran. The study population consisted of 195 stroke patients and 195 controls, selected by the convenience non-random sampling method. Stroke patients had been hospitalized in the neurology ward of the hospital, and controls were selected from hospitalized patients in orthopedic or surgical wards of this center. Controls had no prior history of cerebrovascular accident (CVA) or any neurological disorders. This was an unmatched study.

### Assessment of dietary intake

A validated 168-item food frequency questionnaire (FFQ) was used to assess participants’ usual dietary intake over the previous year. The reliability and validity of FFQ were reported previously in Iranian population [19]. The FFQ contained all foods that were commonly consumed by the Iranian people. The questionnaires were administered by trained and experienced dietitians. Participants were requested to report the frequency of each food item that they were commonly consuming based on a daily, weekly, or monthly intake, by considering the standard portion size for each food item. We calculated the daily frequency for each food item by dividing weekly frequency and monthly frequency to 7 and 30, respectively. Daily portion sizes of consumed foods were converted to grams using household measures. Nutrient intakes from the FFQ were computed through linking the amounts of foods with the Nutritionist IV software, whose nutrient database was based on the USDA food composition table modified for Iranian foods. Total energy intake per day was calculated by summing up energy intakes from all foods. As brain damage or impaired memory is the most common sequel of stroke and recalls of dietary intakes from these patients would be biased, we asked their family members to help complete the FFQ. The reported frequency as well as the portion size was double checked with the person responsible for cooking at home. The food items included in the FFQ were those that had high consumption among Iranian population. Egg consumption was calculated by summing up the consumption of number of boiled egg and processed egg products (including omelet, vegetable cutlets, potato cutlets, and scrambled egg mix).

### Assessment of stroke

In the present study, cases were participants aged 45 years and older, with acute stroke, confirmed by brain computed tomography (CT) or magnetic resonance imaging (MRI). Cases with head trauma, primary intracranial hemorrhage, or subarachnoid or subdural hemorrhage were excluded from the study. The study protocol was approved by local bioethics Review Committee of Isfahan University of Medical Sciences.

### Assessment of other variables

Other required information such as socio-demographic characteristic (like sex, age, education), family medical history, physical activity and smoking status was gathered by the use of questionnaires. Data on physical activity were obtained using the International Physical Activity Questionnaire (IPAQ) and expressed as metabolic equivalent-h/week. Participants were requested to answer the questions about the type of physical activity and its intensity as well as the duration of that activity during the day. The sum of the durations of activities in the physical activity questionnaire had to be 24 h. Data from the physical activity questionnaire were expressed as metabolic equivalents tasks (MET). The validity of this questionnaire was previously reported [20]. Weight was measured using a balanced scale (Seca 700, Germany) and was recorded to the nearest 0.1 kg. Height was recorded using a wall-mounted tape-meter. All participants were able to appropriately stand for their weights and heights to be accurately measured. Weight and height measurement was done in light clothing and barefoot. Body max index (BMI) was calculated as weight (kg) divided by the square of height (m^2^).

### Statistical methods

Statistical analyses were performed by using Statistical Package for Social Science (SPSS Inc., Chicago, IL, USA, version 16.0). All probability values presented are two-tailed, and probability values below 0.05 were considered statistically significant. Independent samples Student’s *t* test was used for comparing means of continuous variables among cases and controls, and chi-square test was applied to compare categorical variables between both groups. We categorized participants based on different levels of egg consumption. General characteristics and dietary intakes of participants across different categories of egg consumption were compared by the use of analysis of variance, chi-square, and analysis of covariance where appropriate. All dietary intakes were obtained by using general linear model with age and total energy intake as covariates. Logistic regression method was used to examine the associations between egg consumption and stroke. Estimates were presented in four models. The first model was adjusted for two non-modifiable risk factors for stroke: age (continuous), sex (categorical), as well as total energy intake (continuous). In the second model, we controlled for two modifiable risk factors for stroke: physical activity (continuous) and smoking (categorical). Additional adjustments were made for dietary intakes (including whole and refined grains, vegetables, low- and high-fat dairy, hydrogenated vegetable oils (HVOs), non-HVOs, fruits, pulses, and total meat all as continuous variables), as other modifiable risk factors, in the third model. Finally, we further adjusted for BMI (continuous) to assess the independent-of-obesity association between egg consumption and stroke. In these step-by-step adjustments, we examined whether the amounts and significances of odds ratios were stable or not. All models were done by treating the first category of egg consumption as the reference. To calculate the trend of odds ratios across increasing quartiles of egg consumption, we considered egg consumption categories as a linear rather than categorical variable.

## Results and discussion

General characteristics of study participants based on the case and control groups as well as across different categories of egg consumption are summarized in Table 1. Individuals with stroke were more likely to be male and older as compared with controls. They had lower BMI and weight and were less likely to be obese as compared with controls. Controls that consumed more than two eggs per week were younger and had lower weight than those who consumed less than one egg per week. Distribution of participants in terms of gender and obesity were not significantly different in controls. Among cases, there was no statistically significant difference in mean age, weight, physical activity, and BMI across different categories of egg consumption. Although distribution of participants in terms of categorical variables was not significantly different among cases, participants in the highest category of egg consumption were more likely to be males (*P* = 0.09) and physically active (*P* = 0.05) compared to those in the lowest category.

**Table 1:** General characteristics of study participants based on cases and controls as well as across different categories of egg consumption

	Cases (*n* = 195)		Controls (*n* = 195)	*P* ^a^
Age (years)	67.9 ± 1.0		61.5 ± 0.8	<0.001
Weight (kg)	69.5 ± 0.9		72.4 ± 1.1	0.05
BMI (kg/m^2^)	25.2 ± 0.3		28.4 ± 1.0	0.01
Male (%)	60		47	0.01
Current smokers (%)	14		6	0.01
Physical activity (MET-min/day)	4766 ± 55.5		3951 ± 49.1	0.43
Obesity^b^ (%)	11		29	<0.001
	Categories of egg consumption (egg/week)			
	<1	1–2	>2	*P* ^c^
Controls (*n* = 195)				
Numbers	81	44	70	
Age (years)	64.3 ± 10.6	62.0 ± 11.7	58.0 ± 8.7	0.01
Weight (kg)	73.4 ± 16.2	70.0 ± 15.8	69.0 ± 11.5	0.03
BMI (kg/m^2^)	30.0 ± 5.2	29.0 ± 6.0	26.2 ± 5.0	0.23
Male (%)	43.2%	47.7%	50.0%	0.70
Physical activity (MET-min/day)	2405.0 ± 5050.2	4181.0 ± 10058.7	5597.7 ± 12655.0	0.12
Obesity^b^ (%)	32.1%	36.4%	21.4%	0.18
Cases (*n* = 195)				
Numbers	117	46	32	
Age (years)	69.3 ± 13.0	65.2 ± 14.7	68.0 ± 13.3	0.22
Weight (kg)	70.0 ± 13.8	69.3 ± 11.2	68.9 ± 13.3	0.91
BMI (kg/m^2^)	25.6 ± 4.4	25.2 ± 3.7	24.3 ± 4.8	0.43
Male (%)	55.6%	60.0%	77.4%	0.09
Physical activity (MET-min/day)	3246.0 ± 5858. 9	6584.8 ± 10670.8	7760.6 ± 20782.1	0.05
Obesity^b^ (%)	14.5%	8.9%	3.2%	0.18

Data are means ± SD, unless indicated

^a^Obtained by the use of independent samples Student’s *t* test for continuous variables and chi-square for categorical variables

^b^Obesity, BMI ≥30 kg/m^2^

^c^Obtained by the use of ANOVA for continuous variables and chi-square for categorical variables

Dietary intakes of participants based on the case and control groups as well as across different categories of egg consumption are shown in Table 2. Controls that were eating more than two eggs per week had higher intakes of vegetables (383.5 ± 225.4 vs. 222.7 ± 222.4 g/day, *P* < 0.001) than those who consumed less than one egg per week. No other significant difference was seen in food and nutrient intakes comparing different categories of egg consumption. Stroke patients that had consumed more than two eggs per week had greater intakes of high-fat dairy, nuts, and legumes compared with those who had consumed less than one egg per week; however, no other statistically significant difference was observed in mean total energy, nutrients, and food group consumption comparing different categories of egg consumption.

**Table 2:** Dietary intakes of study participants based on cases and controls as well as across different categories of egg consumption

	Cases (*n* = 195)		Controls (*n* = 195)	*P* ^a^
Egg consumption (*n*)				0.01
<1	117		81	
1–2	46		44	
>2	32		70	
Total energy (kcal/day)	2074 ± 71		2110 ± 63	0.70
Grain (g/day)	292 ± 12		326 ± 14	0.07
Vegetables (g/day)	247 ± 12		285 ± 16	0.06
Red and processed meat (g/day)	48 ± 16		28 ± 4	0.21
Low-fat dairy (g/day)	272 ± 15		340 ± 20	0.01
High-fat dairy (g/day)	132 ± 15		73 ± 9	<0.001
Hydrogenated vegetable oils (g/day)	22 ± 3		21 ± 2	0.75
Non-hydrogenated vegetable oils (g/day)	10 ± 1		19 ± 1	<0.001
Fruits (g/day)	360 ± 30		281 ± 17	0.02
Nuts and legumes (g/day)	46 ± 3		38 ± 3	0.04
	Categories of egg consumption (egg/week)			
	<1	1–2	>2	*P* ^a^
Controls (*n* = 195)				
Numbers	81	44	70	
Total energy (kcal/day)	2062.4 ± 903.3	2066.3 ± 833.3	2192.2 ± 839.8	0.62
Grain (g/day)	321.8 ± 202.9	342.7 ± 221.0	319.7 ± 157.7	0.80
Vegetables (g/day)	222.7 ± 222.4	244.0 ± 154.98	383.5 ± 225.4	<0.001
Red and processed meat (g/day)	32.3 ± 72.7	17.9 ± 13.7	28.2 ± 33.4	0.33
Low-fat dairy (g/day)	324.3 ± 308.0	301.2 ± 222.3	382.1 ± 278.0	0.26
High-fat dairy (g/day)	59.8 ± 103.5	70.8 ± 103.5	91.2 ± 154.4	0.30
Hydrogenated vegetable oils (g/day)	21.2 ± 33.4	19.8 ± 27.7	15.0 ± 26.2	0.43
Non-hydrogenated vegetable oils (g/day)	21.9 ± 22.6	19.9 ± 13.7	15.6 ± 12.2	0.09
Fruits (g/day)	264.3 ± 267.2	229.3 ± 158.2	331.2 ± 243.7	0.06
Nuts and legumes (g/day)	32.3 ± 28.2	35.2 ± 33.8	45.3 ± 44.6	0.08
Cases (*n* = 195)				
Numbers	117	46	32	
Total energy (kcal/day)	1972.0 ± 1042.2	2235.4 ± 895.6	2232.7 ± 835.6	0.20
Grain (g/day)	290.9 ± 154.1	308.4 ± 214.5	275.2 ± 140.4	0.69
Vegetables (g/day)	232.0 ± 153.8	268.7 ± 222.2	273.6 ± 157.0	0.32
Red and processed meat (g/day)	56.4 ± 280.7	35.3 ± 38.2	35.5 ± 31.0	0.81
Low-fat dairy (g/day)	284.7 ± 205.5	255.8 ± 225.7	249.9 ± 192.2	0.59
High-fat dairy (g/day)	102.4 ± 181.5	182.7 ± 259.9	171.9 ± 217.5	0.05
Hydrogenated vegetable oils (g/day)	16.2 ± 35.2	23.8 ± 37.2	22.1 ± 38.4	0.43
Non-hydrogenated vegetable oils (g/day)	10.2 ± 10.2	10.4 ± 11.2	9.9 ± 11.1	0.98
Fruits (g/day)	347.2 ± 428.0	415.9 ± 461.2	329.8 ± 254.3	0.58
Nuts and legumes (g/day)	40.5 ± 48.2	44.4 ± 34.8	72.0 ± 54.8	0.01

Data are means ± SD. Data for energy intake are adjusted for age. Data for other dietary variables are adjusted for age and total energy intake

^a^Obtained by the use of ANCOVA

Multivariable-adjusted odds ratios (ORs) and 95% CIs for stroke across different categories of egg consumption are provided in Table 3. Consumption of more than two eggs per week was associated with lower odds of stroke, such that those in the highest category of egg intake were 77% lower odds to have stroke (OR 0.23; 95% CI 0.11–0.45), compared with those with the lowest intake, after taking potential confounders, including dietary intakes, into account. Further controlling for BMI strengthened the association (OR 0.20; 95% CI 0.09–0.41). After adjustments for potential confounders in models 1 to 4, ORs increased in the second category of egg consumption (1–2 eggs/day), while ORs in the third category of egg consumption (>2 eggs/day) decreased.

**Table 3:** Multivariate-adjusted odds ratio for stroke comparing cases and controls with respect to egg consumption

	Categories of egg consumption (egg/week)			
	<1	1–2	>2	*P*_trend_^a^
Crude	1.00	0.70 (0.42–1.17)	0.30 (0.18–0.51)	0.034
Model 1^b^	1.00	0.75 (0.44–1.30)	0.33 (0.19–0.57)	0.036
Model 2^c^	1.00	0.74 (0.43–1.29)	0.33 (0.19–0.58)	0.037
Model 3^d^	1.00	0.78 (0.41–1.50)	0.23 (0.11–0.45)	0.040
Model 4^e^	1.00	0.79 (0.41–1.50)	0.20 (0.09–0.41)	0.051

Data are odds ratio (95% confidence interval)

^a^Obtained by the use of categories of egg consumption as a linear rather than categorical variable

^b^Adjusted for age, sex, and energy intake (nuts and legumes, red and processed meats, grains, vegetables, SSB, low-fat dairy, high-fat dairy, hydrogenated vegetable oils, non-hydrogenated vegetable oils, and fruits)

^c^Further control for physical activity and smoking

^d^Additionally adjusted for dietary intakes

^e^Further adjustment for BMI

In the present study, we found an inverse significant association between high consumption of eggs in long term and risk of stroke among a group of hospitalized Iranian adults. This relationship remained significant even after controlling for several potential confounders. To our knowledge, this is the first study that has reported an association between egg intake and risk of stroke from a Middle Eastern population.

Stroke has been among the leading causes of disability in Middle Eastern countries, where the traditional dietary patterns include high intake of refined carbohydrates [21]. In addition, consumption of eggs in these countries is lower than that in the USA and Europe [7, 18]. Our findings in the present study are consistent with earlier findings in other countries [14, 15, 17]. In a prospective study in US adults, a significant inverse association was observed between high egg intake and risk of mortality from stroke [14]. In a large-scale population-based cohort study in Japan, consumption of animal products, including eggs, was seen to be protectively associated with the risk of stroke [17]. In contrast to our findings, some studies have failed to reach a significant association between egg consumption and risk of stroke [15, 18]. Qureshi et al. in a nationally representative cohort of 9734 adults found that consumption of greater than six eggs per week did not change the risk of stroke and ischemic stroke [15]. Different findings might be explained by the different study designs, categorization of egg intake, different dietary assessment tools applied in the studies, as well as controlling for different confounders. In addition, type of stroke might also provide some explanations for the discrepant findings.

In the present study, we asked the participants to report their usual dietary intake over the last year; however, the association we have observed between egg consumption and stroke might reflect the long term intake of egg (prior to the previous 1 year), rather than the last year examined; this point should be taken into account while interpreting the findings.

The mechanisms through which egg consumption might influence the risk of stroke are unknown. The proposed ones include its high content of vitamins E, B_12_, and B_6_ and folate. Earlier publications have demonstrated an inverse association between dietary intakes of folate, vitamin B_12_, and B_6_ and risk of stroke. These nutrients might influence the risk through reducing serum levels of homocysteine and as a result improving endothelial function [22, 23]. In addition, high protein content of eggs might be involved in its protective association with stroke through lowering blood pressure [17]. Vitamin E has also been demonstrated to have protective effects on stroke through reducing oxidative stress and suppression of thrombosis [4].

Our study has several limitations. The case–control design of the study would not allow us to infer causality. Although we used a validated FFQ for dietary intake assessment, misclassification of participants may occur. Although we controlled the analysis for several confounding variables, the existence of residual confounding cannot be excluded. Furthermore, random errors, as in all epidemiologic studies, might affect our results, because diet and lifestyle information might be collected with some degree of errors. The two types of strokes (ischemic and hemorrhagic) have different risk factors, and earlier data have shown that the relation between dietary factors and stroke risk might be different for ischemic and hemorrhagic strokes [24]. As most cases in the current study were hemorrhagic cases (*n* = 176) and the small number of ischemic strokes was available (*n* = 19), we analyzed all data together. The existence of recall bias and selection bias in case–control studies must also be taken into account. This is particularly relevant for our study because we enrolled newly diagnosed cases of stroke, and the interviewing close to the time of diagnosis may increase the potential for recall bias. However, some studies in the field of nutritional epidemiology, which have assessed the effect of recall bias on the overall study results, reported that this bias was minor and negligible [19, 25]. Selection bias results from inappropriate enrolment of controls. Typical control individuals should represent the population from which the cases arose; however, in the current study, control participants have been selected from orthopedic and surgery wards which, in turn, would result in potential selection bias. Although the response rate of the controls was high (93%), the possibility of slight selection bias from non-responders cannot be excluded. As the amount of eggs contained in foods such as ice cream and cakes are little, we did not quantify them. In addition, data for cases were collected from family members as well as the cases themselves, while data for controls were not collected from their families; this different procedure might introduce a potential source of bias and measurement error. Finally, it must be considered that the controls were not strictly matched to cases and several differences between these two groups might contribute to the associations reported.

## Conclusions

Given the abovementioned limitations, we found evidence indicating that high intake of eggs during the past 1 year was associated with a lower risk of stroke. Further studies, in particular prospective studies in the Middle Eastern population, are required to confirm these findings.

## Abbreviations

95% CI: 95% confidence interval
AMD: Age-related macular degeneration
BMI: Body mass index
CHD: Coronary heart disease
CT: Computed tomography
CVA: Cerebrovascular accident
FFQ: Food frequency questionnaire
HVOs: Hydrogenated vegetable oils
IPAQ: International Physical Activity Questionnaire
IUMS: Isfahan University of Medical Sciences
MET: Metabolic equivalents tasks
MPOD: Macular pigment optical density
MRI: Magnetic resonance imaging
non-HVOs: Non-hydrogenated vegetable oils
NTD: Neural tube defects
OR: Odds ratio
SPSS: Statistical package for social science
